# Effect of IGC‐AD1 on Psychosis in Alzheimer’s Disease: Results from a Phase 1 MAD Trial

**DOI:** 10.1002/alz.095593

**Published:** 2025-01-09

**Authors:** Maria Juanita Arbelaez, Evelyn Gutiérrez, Margarita Venegas, Maria Alejandra Tangarife, Laura Tatiana Sanchez, Saadia Shahnawaz, William Julio De Jesus, Karen Y Pujals, Claudia Grimaldi, Jagadeesh S Rao, Elliot Hong, Ram Mukunda

**Affiliations:** ^1^ IGC Pharma, Bogotá, Bogotá D.C. Colombia; ^2^ IGC Pharma, Potomac, MD USA; ^3^ Santa Cruz Behavioral, Bayamon, PR USA; ^4^ UTHealth Houston McGovern Medical School, Houston, TX USA

## Abstract

**Background:**

We present Phase 1 trial data using the Neuropsychiatric Inventory (“NPI”) domains, NPI‐delusions and NPI‐hallucinations as symptoms of psychosis in participants with Alzheimer’s (“AD”) receiving IGC‐AD1, a combination of low concentration delta 9‐tetrahydrocannabinol (“THC”) and melatonin. Cannabis use is considered an established risk factor for psychosis in young people. Psychosis is prevalent in AD patients, with around 50% experiencing it^1^, generating safety concerns regarding the use of THC in these patients.

**Method:**

13 patients (81.5±5 years, 69.2% female) diagnosed with Alzheimer’s disease were included in a three‐cohort, MAD, phase‐1 safety, and tolerability trial (IND146069, NCT04749563). In Cohorts‐1, 2, and 3, one milliliter of IGC‐AD1 was administered QD, BID, TID, respectively, for 14‐days (“EOT”), with a minimum washout period of 4‐days between cohorts. In each cohort, there were active participants with delusions/hallucinations at baseline (Cohort‐1, n = 5; Cohort‐2, n = 2; Cohort‐3, n = 3). In Cohort‐2, one placebo experienced hallucinations on Day 10. Descriptive statistics will summarize the data. Paired T‐test was applied to compare the difference between Baseline‐D10 and Baseline‐EOT for each cohort (SPSSv.28). Solicited/unsolicited Adverse Events and vitals were monitored daily.

**Result:**

Reductions in NPI‐delusions and NPI‐hallucinations were observed from baseline to Day 10 (delusions: ‐1.8, 27%, p = 0.18; hallucinations: ‐1, 18%, p = 0.446), with greater reductions noted at EOT (delusions: ‐3.8, 58%, p = 0.038; hallucinations: ‐3.4, 61%, p = 0.062). Cohort‐2: Decreases were observed in both NPI‐delusions and NPI‐hallucinations at D10 (delusions: ‐3.5, 100%, p = 0.09; hallucinations: ‐1.5, 25%, p = 0.205), with more decreases observed at EOT (delusions: ‐2, 57%, p = 0.295; hallucinations: ‐4, 67%, p = 0.157). Cohort‐3: Changes in NPI‐delusions and NPI‐hallucinations were observed at Day 10 (delusions: ‐3, 69%, p = 0.035; hallucinations: 2.33, 87%, p = 0.192), with improvements noted at EOT for NPI‐delusions (mean difference = ‐4.33, 100%, p = 0.145) and no change in NPI‐hallucinations (mean difference = 0, p = 1). No serious adverse events were reported, and there were no major changes in concomitant medications.

**Conclusion:**

IGC‐AD1 was safe and well‐tolerated, without causing or worsening delusions/hallucinations as evaluated by the NPI. Contrary to other findings related to cannabis use, we observed clinical reduction approaching, in some cases, statistical significance. The signals warrant further analysis in a larger trial.

